# Effect modification of polypharmacy on incident frailty by chronic kidney disease in older adults

**DOI:** 10.1186/s12877-024-04887-5

**Published:** 2024-04-12

**Authors:** Nina Mielke, Muhammad Helmi Barghouth, Anne-Katrin Fietz, Cédric Villain, Tim Bothe, Natalie Ebert, Elke Schaeffner

**Affiliations:** 1 Institute of Public Health, Charité– Universitätsmedizin Berlin, Charitéplatz 1, 10117 Berlin, Germany; 2 Institute of Biometry and Clinical Epidemiology, Charité– Universitätsmedizin Berlin, Charitéplatz 1, 10117 Berlin, Germany; 3 Service de Gériatrie, Normandie Univ UNICAEN, INSERM U1075 COMETE, CHU de Caen, Caen, France

**Keywords:** Epidemiology, Medication, Community-dwelling cohort, CKD, Age, Frailty, Polypharmacy

## Abstract

**Background:**

Frailty and polypharmacy are common conditions in older adults, especially in those with chronic kidney disease (CKD). Therefore, we analyzed the association of polypharmacy and incident frailty and the effect modification by CKD in very old adults.

**Methods:**

In non-frail individuals within the Berlin Initiative (cohort) Study, polypharmacy (≥ 5 medications) was assessed according to multiple definitions based on the number of regular and on demand prescription and over the counter drugs, as well as vitamins and supplements. CKD was defined as an estimated glomerular filtration rate < 60 mL/min/1.73m^2^ and/or an albumin-creatinine ratio ≥ 30 mg/g. Incident frailty was assessed at follow-up using Fried criteria. Logistic regression was applied to assess (1) the association of different polypharmacy definitions with incident frailty and (2) effect modification by CKD.

**Results:**

In this cohort study, out of 757 non-frail participants (mean age 82.9 years, 52% female, 74% CKD), 298 (39%) participants reported polypharmacy. Over the observation period of 2.1 years, 105 became frail. Individuals with polypharmacy had 1.96 adjusted odds (95% confidence interval (CI): 1.20–3.19) of becoming frail compared to participants without polypharmacy. The effect of polypharmacy on incident frailty was modified by CKD on the additive scale (relative excess risk due to interaction: 1.56; 95% CI 0.01–3.12).

**Conclusions:**

This study demonstrates an association of polypharmacy and incident frailty and suggests strong evidence for an effect modification of CKD on polypharmacy and incident frailty. Revision of prescriptions could be a target strategy to prevent frailty occurrence, especially in older adults with CKD.

**Supplementary Information:**

The online version contains supplementary material available at 10.1186/s12877-024-04887-5.

## Introduction

Chronic diseases, acute events, and the need for symptom management increase with age, which is reflected in the concurrent intake of multiple medications [1]. This phenomenon is commonly referred to as polypharmacy and is especially frequent in older adults [2]. However, there is no official consensus on the definition of polypharmacy [3]. The most common definition is the use of at least five medications, often without further specification as to prescription requirement (i.e. prescription or over-the-counter (OTC)) or pattern of intake (i.e. regular or on-demand). Polypharmacy itself is associated with adverse events such as falls, hospital admissions, and mortality [2].

The prevalence of frailty also increases with age [4]. Frailty is described as a biological syndrome with accelerated decline in physiological reserves and resilience to stressors, also resulting in increased risk of similar adverse outcomes such as risk of falls, hospitalization, disability in activities of daily living, need of nursing home, or mortality [5, 6]. Studies analyzing cross-sectional data mostly found an association between polypharmacy and frailty but the temporal relationship between both remains unclear [7]. Longitudinal studies on polypharmacy and incident frailty have been inconclusive [8–13] and sparse [14]. A study from Germany showed that in older adults with a mean age of 70 years, polypharmacy was associated with a 1.5 odds for incident frailty [9].

Both the incidence of frailty as well as polypharmacy are associated with another globally prevalent health burden affecting older adults, namely chronic kidney disease (CKD) [15–17]. Prevalence of CKD varies from one third to two thirds in the adult population over the age of 75 years in Europe [18]. Older individuals with CKD often have a high prevalence of comorbidities and therefore polypharmacy is inevitable [19, 20]. The complex treatment regimens in individuals with CKD for example increase the potential for adverse drug-drug interactions and consequently, adverse side effects [15, 21].

In order to disentangle the unclear relationship between polypharmacy, frailty, and CKD, we hypothesize that in very old adults (1) different definitions of polypharmacy alter the effect estimation on incident frailty and (2) that CKD modifies the effect of polypharmacy on incident frailty.

## Method

### Study population

**Fig. 1:**
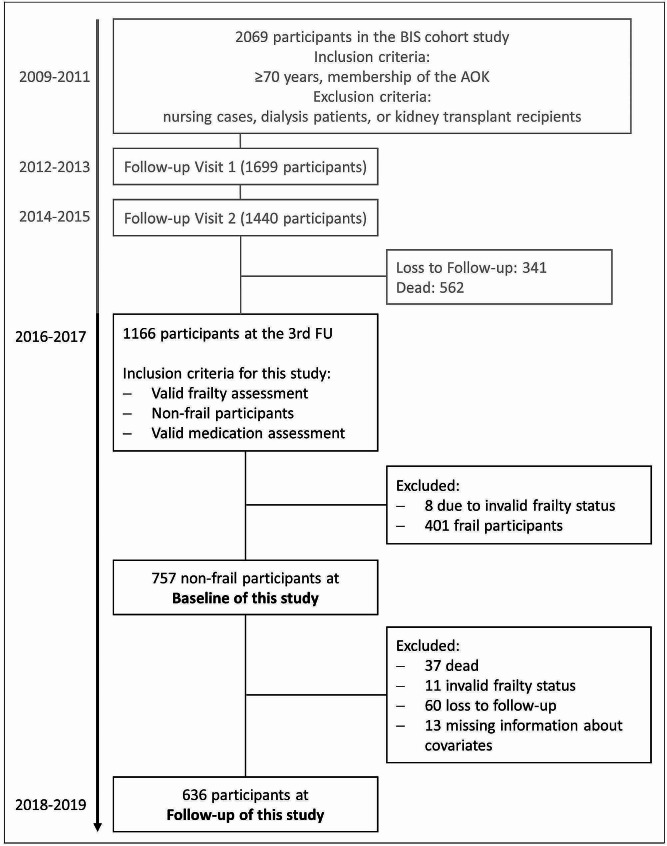
Overview of the Berlin Initiative Study (BIS) population. The flowchart shows the composition of the study population. The light gray section of the figure indicates the part of the BIS study that preceded this study. Frailty assessment was implemented at the 3rd follow-up (FU) of the BIS, defining the baseline visit for this study. Exclusion and inclusion criteria are displayed as well as the excluded participants for the regression analysis AOK: statutory health insurance fund AOK Nordost– Die Gesundheitskasse

The Berlin Initiative Study (BIS) is a cohort of 2069 community-dwelling older adults. Face-to-face study visits were conducted biennially using the infrastructure of 16 private nephrology practices and outpatient clinics in Berlin, Germany [22, 23]. Briefly, inclusion criteria were a minimum age of 70 years and membership of the statutory health insurance fund *AOK Nordost– Die Gesundheitskasse* (AOK). Exclusion criteria at BIS baseline were nursing cases, dialysis patients, or kidney transplant recipients. Participants were enrolled between November 2009 and July 2011 and written informed consent was obtained. At the third follow-up visit (2016–2017) during which frailty was first assessed, 1166 participants could be re-interviewed. Therefore this visit will be referred to as the baseline of the present study. To be included in the present study participants had to have a valid medication and frailty assessment. Eight participants were excluded due to non-valid frailty assessment. Furthermore, to investigate the outcome incident frailty, participants with prevalent frailty were also excluded (*n* = 401) leaving 757 non-frail (robust or prefrail) participants to be included in the present study (Fig. 1). The study was approved by the ethics committee, Charité - Universitätsmedizin Berlin, Germany (EA2/009/08) and is in accordance with the 1964 Helsinki declaration and its later amendments.

### Exposure: polypharmacy assessment

To obtain as complete medication information as possible, all participants were asked to bring their medication lists as well as all medication packages to the visits. At each visit, medically trained staff then conducted the medication assessment. All regular and on-demand prescriptions as well as OTC medications in addition to supplements, vitamins, and minerals were assessed based on the packages, lists, and the participants’ self-report, and entered into a standardized computer-based questionnaire [24]. The questionnaire was linked to a drug database and automatically provided information such as the Anatomical Therapeutic Chemical (ATC) code and prescription requirement. The primary definition of polypharmacy was based on the number of regular prescription drugs (*regular prescription polypharmacy*). Secondary definitions of polypharmacy were as follows: *active substance polypharmacy*, defined as the number of active substances in regular prescription drugs accounting for the presence of more than one active substance in combination drugs; *regular polypharmacy*, defined as the number of regular prescription and regular OTC drugs; *regular and on-demand polypharmacy*, additionally including the number of on-demand prescription and on-demand OTC drugs, and *all polypharmacy*, defined as all medications including the number of vitamins, minerals, and supplements. For all definitions, no polypharmacy was defined as taking of 0–4 drugs, polypharmacy as taking of 5–9 drugs, and hyperpolypharmacy as taking of ≥ 10 drugs.

### Outcome: incident frailty

Frailty status was assessed using the modified Fried criteria [25]: shrinking, exhaustion, weakness (adapted from Fried without modifications), low physical activity (corresponds to engaging in physical activity, e.g., brisk walking, that exceeds 30 min less than once a week), and slowness (15 s or more in the Timed Up and Go test [26]). Participants were defined as frail if they fulfilled ≥ 3 of the above criteria or non-frail if they were prefrail (1–2 criteria fulfilled) or robust (no criterion fulfilled). At baseline, only non-frail participants were eligible. At the following study visit, the frailty status was re-assessed and participants meeting at least three of the five frailty criteria were defined as incident frail.

### Covariable assessment

At each study visit, a standardized computer-based questionnaire was used to collect data on demographics, lifestyle variables, and morbidity. Anthropometric and geriatric assessments were also conducted. The primary study data were complemented by AOK health claims data linked on person-level. This allowed supplementing self-reported data on, e.g., morbidities coded according to the 10th Revision of the International Statistical Classification of Diseases and Related Health Problems (ICD-10), or information from participants who were no longer followed up. The following covariates were derived from BIS data at baseline: age, gender, the short version of the CASMIN (Comparative Analysis of Social Mobility in Industrial Nations) classification of education [27], marital status (single, married, divorced, widowed), smoking status (never, ever) and body mass index (BMI) (< 22, 22-<30, or ≥ 30 kg/m^2^). CKD was defined as having an estimated glomerular filtration rate (eGFR) < 60 mL/min/1.73m^2^ based on the BIS2 equation [28] and albuminuria defined as an albumin-creatinine ratio (ACR) ≥ 30 mg/g. In line with the Kidney Disease Improving Global Outcomes (KDIGO) guidelines [29], kidney disease was found to be chronic in at least 97% of the participants (Supplement A). The Charlson Comorbidity Index (CCI) [30] was used as a measure of morbidity in the regression analyses and was compiled from the AOK health claim data.

### Statistical analyses

The baseline characteristics of the study population are described in total as well as stratified by categories of the primary polypharmacy definition. Descriptive analysis included absolute and relative frequencies for categorical variables and for continuous variables, depending on their distribution, either mean with standard deviation (SD) or median with interquartile range (IQR).

The ten most frequent medication groups on the therapeutic subgroup level (3-digit ATC code) were assessed for each category of the primary polypharmacy definition (*regular prescription polypharmacy*). Within these groups, the five most prescribed medications were identified on the chemical substance level (7-digit ATC code).

Participants who were lost to follow-up (*n* = 60), had died before the follow-up visit (*n* = 37), did not have a valid frailty assessment at the follow-up visit (*n* = 11), or had missing data regarding covariables (*n* = 13) and thus were excluded from the regression analyses. To evaluate potential selection bias due to excluding these participants in the regression models, baseline characteristics by inclusion status were compared. To also address the potential competing risk by death, we compared mortality by exposure strata. Logistic regression models were used to analyze the association of polypharmacy and incident frailty to estimate crude and adjusted odds ratios (OR) and corresponding 95% confidence intervals (CI). The adjusted models included the following baseline variables determined by directed acyclic graphs [31]: age, gender, smoking, CASMIN, marital status, BMI, and CCI. The CCI was also used to address confounding by indication [32]. These analyses were then repeated with all other polypharmacy definitions in an exploratory approach. To allow comparison with studies that assessed polypharmacy as a dichotomous variable (no polypharmacy vs. polypharmacy), all models were repeated using this approach in a sensitivity analysis.

Effect Modification by CKD was investigated on multiplicative and additive scales as suggested by Knol and VanderWeele [33] to show to what extent the joint effect of exposures differs from the separate effects. By calculating effect estimates across both strata of the effect modifier with one reference category, this allows the identification of the subpopulation with the highest risk of experiencing the outcome. Furthermore, it allows the determination of interaction on the additive scale which is more relevant within the public health context [34]. Reporting effect modification on both scales is also recommended according to the Strengthening the Reporting of Observational Studies in Epidemiology (STROBE) statement [35].

Since the number of events in the no CKD group was low, the polypharmacy and hyperpolypharmacy categories were collapsed and a logistic regression model adjusted for age, gender, smoking, CASMIN, marital status, BMI, and CCI was applied. Model-adjusted risks with corresponding 95% CI and measures of effect modification on both multiplicative (Ratio of ORs) and additive (Relative Excess Risk due to Interaction, RERI) scales were computed using the InteractionR package [36]. In addition, logistic regression analyses were repeated after addition of CKD as an interaction term as well as stratified by CKD.

All statistical analyses were conducted with R (Version 4.1.1; R Foundation for Statistical Computing, Vienna, Austria) and reporting of results was performed according to the STROBE statement (Supplement B).

## Results

### Characteristics of the study population

The characteristics of the study participants at baseline are displayed in Table 1. Of 757 participants with a mean (SD) age of 82.9 (4.9) years 52.4% were female. With respect to regular prescription polypharmacy, 276 (36.5%) participants took 5–9 prescribed medications regularly and 22 (2.9%) participants took 10 or more prescribed medications regularly. With a higher number of medications, individuals had more often comorbidities (CCI; 4 vs. 10).

**Table 1:** Main characteristics of the non-frail study population by regular prescription polypharmacy at baseline

	Total	No polypharmacy	Polypharmacy	Hyperpolypharmacy
*n*	757	459	276	22
Age in years, mean (SD)	82.9 (4.9)	82.9 (5.0)	82.9 (4.7)	81.7 (3.6)
Gender, n (%)				
Female	397 (52.4)	250 (54.5)	135 (48.9)	12 (54.5)
CASMIN, n (%)				
Low	450 (59.4)	263 (57.3)	173 (62.7)	14 (63.6)
Middle	147 (19.4)	94 (20.5)	50 (18.1)	3 (13.6)
High	157 (20.7)	101 (22.0)	51 (18.5)	0
Missing	3 (0.4)	1 (0.2)	2 (0.7)	5 (22.7)
Marital Status, n (%)				
Married	395 (52.2)	234 (51.0)	146 (52.9)	15 (68.2)
Single	35 (4.6)	23 (5.0)	12 (4.3)	0
Divorced	62 (8.2)	45 (9.8)	17 (6.2)	0
Widowed	265 (35.0)	157 (34.2)	101 (36.6)	7 (31.8)
Smoking, n (%)				
Ever	354 (46.8)	199 (43.4)	140 (50.7)	15 (68.2)
BMI in kg/m^2^, n (%)				
< 22	72 (9.5)	57 (12.4)	15 (5.4)	0
22-<30	535 (70.7)	329 (71.7)	189 (68.5)	17 (77.3)
≥30	150 (19.8)	73 (15.9)	72 (26.1)	5 (22.7)
CCI, median [IQR]	5 [3–7]	4 [2–6]	6 [5–8]	10 [7–11]
Missing, n (%)	11 (1.5)	8 (1.7)	3 (1.1)	0
CKD, n (%)	557 (73.6)	312 (68.0)	224 (81.2)	21 (95.5)
Missing	11 (1.5)	8 (1.7)	3 (1.1)	0
eGFR_BIS2_				
mean (SD)	52.9 (12.6)	55.4 (11.5)	49.5 (13.0)	40.5 (13.1)
Missing, n (%)	4 (0.5)	3 (0.7)	0	1 (4.5)
ACR, n (%)				
≥ 30 mg/g	176 (23.2)	89 (19.4)	81 (29.3)	6 (27.3)
Missing	19 (2.5)	12 (2.6)	6 (2.2)	1 (4.5)

BMI: Body mass index; CCI: Charlson Comorbidity Index, CKD: Chronic kidney disease defined as eGFR_BIS2_ <60 mL/min/1.73m^2^ and/or albuminuria defined as ACR ≥ 30 mg/g.; eGFR_BIS2_: Estimated glomerular filtration rate based on the BIS2 equation; ACR: Albumin-creatinine ratio; CASMIN: short version of the Comparative Analysis of Social Mobility in Industrial Nations classification of education; IQR: Interquartile range; No missing values for age, gender, marital status, smoking, BMI

### Most frequent medication categories by polypharmacy status

**Fig. 2:**
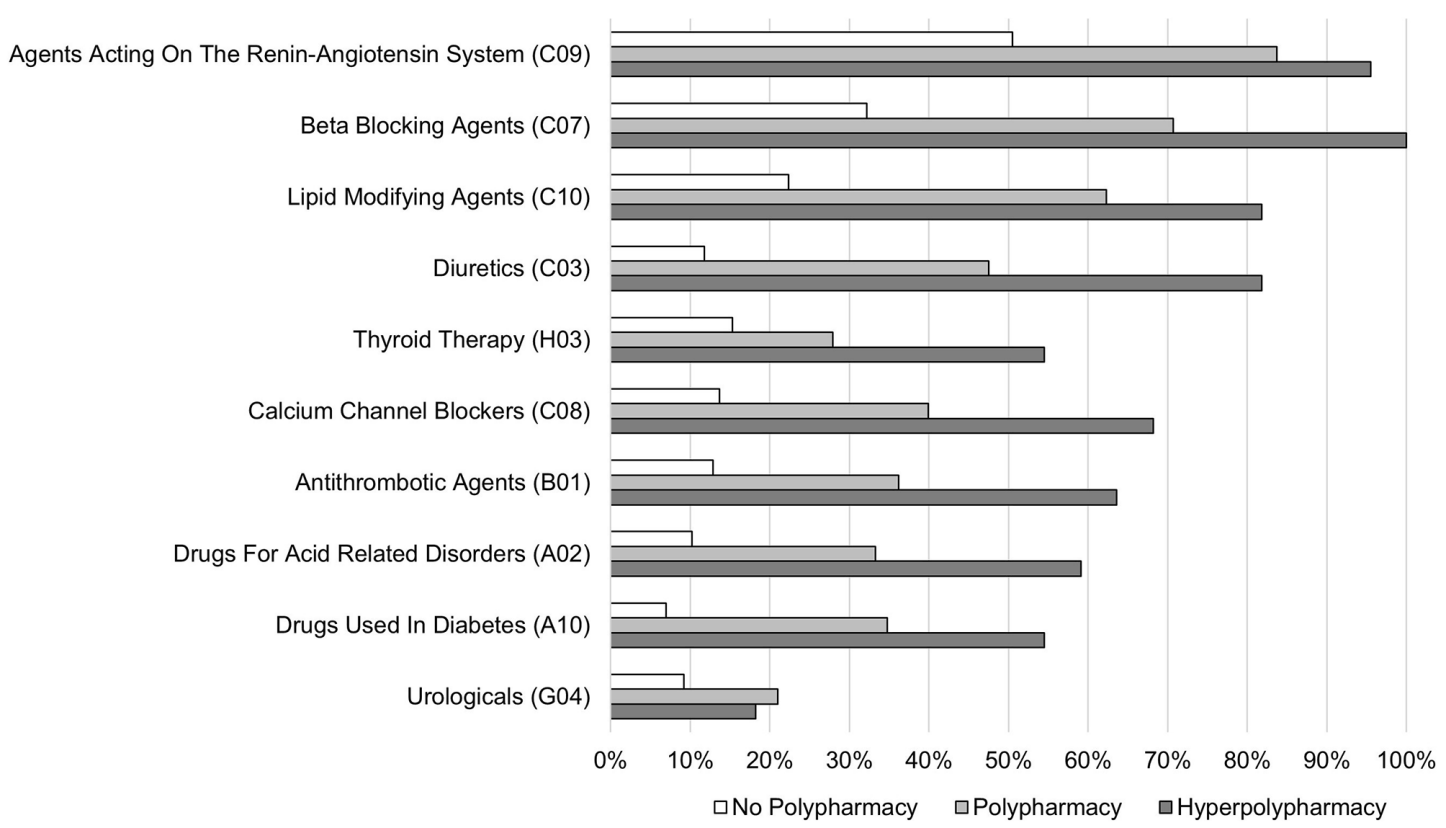
Ten most frequently prescribed drugs on the therapeutic subgroup level (3-digit ATC codes) by polypharmacy categories

Half of the ten most prescribed medication groups were drugs acting on the cardiovascular system (Fig. 2). The most frequent medication group was agents acting on the renin-angiotensin system (ATC C09), followed by beta blocking agents (ATC C07) and lipid modifying agents (C10), regardless of the polypharmacy category. However, the prevalence varied substantially between the different polypharmacy categories. Almost every participant with hyperpolypharmacy was taking a medication in the ATC group C09 or C07 compared to 50% or 32%, respectively, of the participants without polypharmacy. The five most prescribed substances in each group did not vary substantially between polypharmacy groups (Supplement Table 1).

### Polypharmacy and incident frailty

After a median follow-up of 2.1 (2.0-2.3) years (IQR), 105 out of 757 participants (13.9%) became frail and 37 (4.9%) died. Most participants who died had polypharmacy, hyperpolypharmacy or CKD (Supplement Tables 2 and 3). Individuals with regular prescription polypharmacy (22.5%) and hyperpolypharmacy (27.8%) became more often frail compared to individuals without polypharmacy (12.1%).

For the regression analyses, participants with missing data were excluded. The baseline characteristics of the non-frail study population were very similar across inclusion status (Supplement Table 4).

Individuals with regular prescription polypharmacy had higher odds (adjusted OR 1.96; 95% CI 1.20–3.19) of becoming frail compared to participants without polypharmacy (Table 2). The odds of becoming frail was even higher for those with hyperpolypharmacy (OR adjusted 2.87; 95% CI 0.85–9.69).

The results of dichotomous polypharmacy categorization demonstrated effect estimates that were similar to the polypharmacy category of ordinal categorization (Supplement Table 5).

**Table 2:** Association of polypharmacy with incident frailty

	Total	Number of participants with incident frailty (%)	Crude Model OR (95% CI)	Adjusted Model* OR (95% CI)
**Regular Prescription Polypharmacy**				
No Polypharmacy	389	46 (11.8)	Reference	Reference
Polypharmacy	229	51 (22.3)	2.14 (1.38–3.31)	1.96 (1.20–3.19)
Hyperpolypharmacy	18	5 (27.8)	2.87 (0.98–8.41)	2.87 (0.85–9.69)
**Active Substance Polypharmacy**				
No Polypharmacy	367	44 (12.0)	Reference	Reference
Polypharmacy	238	49 (20.6)	1.90 (1.22–2.97)	1.73 (1.06–2.81)
Hyperpolypharmacy	31	9 (29.0)	3.00 (1.30–6.94)	2.86 (1.11–7.39)
**Regular Polypharmacy**				
No Polypharmacy	319	39 (12.2)	Reference	Reference
Polypharmacy	277	48 (17.3)	1.51 (0.95–2.38)	1.32 (0.79–2.21)
Hyperpolypharmacy	40	15 (37.5)	4.31 (2.09–8.87)	4.08 (1.70–9.81)
**Regular and On-Demand Polypharmacy**				
No Polypharmacy	261	29 (11.1)	Reference	Reference
Polypharmacy	302	52 (17.2)	1.66 (1.02–2.71)	1.56 (0.91–2.67)
Hyperpolypharmacy	73	21 (28.8)	3.23 (1.71–6.11)	3.01 (1.43–6.37)
**All Polypharmacy**				
No Polypharmacy	207	22 (10.6)	Reference	Reference
Polypharmacy	309	48 (15.5)	1.55 (0.90–2.65)	1.45 (0.81–2.59)
Hyperpolypharmacy	120	32 (26.7)	3.06 (1.68–5.57)	3.03 (1.52–6.05)

OR: odds ratio; 95% CI: 95% confidence interval; *regular prescription polypharmacy*: number of regular prescription drugs; *active substance polypharmacy*:number of active substances in regular prescription drugs accounting for more than one active substance in combination drugs; *regular polypharmacy*: number of regular prescription and regular OTC drugs; *regular and on-demand polypharmacy*: additionally including the number of on-demand prescription and on-demand OTC drugs; *all polypharmacy*: all drugs including the number of vitamins, minerals, and supplements. For all definitions, no polypharmacy was defined as taking of 0–4 drugs, polypharmacy as taking of 5–9 drugs, and hyperpolypharmacy as taking of ≥ 10 drugs

*Adjusted for age, gender, smoking, short version of the Comparative Analysis of Social Mobility in Industrial Nations classification of education, marital status, Charlson Comorbidity Index, and Body mass index

### Effect estimation using different polypharmacy definitions

The association of different polypharmacy definitions with incident frailty is displayed in Table 2. The association of polypharmacy and incident frailty was slightly attenuated when the polypharmacy definition of active substances was used: active substance polypharmacy (OR 1.73; 95% CI 1.06–2.81) vs. regular prescription polypharmacy (OR 1.96; 95% CI 1.20–3.19) and for hyperpolypharmacy (OR 2.86; 95% CI 1.11–7.39) vs. (OR 2.87; 95% CI 0.85–9.69), respectively. Including regular OTC drugs in the polypharmacy definition (regular polypharmacy) attenuated the relative number of participants with incident frailty among individuals with polypharmacy (17.3 vs. 22.3%) and elevated it for individuals with hyperpolypharmacy (37.5 vs. 27.8%). Using this regular polypharmacy definition, the adjusted odds of becoming frail for individuals with hyperpolypharmacy was 4.08 (95% CI 1.70–9.81) compared to individuals without polypharmacy. Subsequently, the polypharmacy definition was broadened to include on-demand medications. Both the distribution of incident frail individuals by polypharmacy category as well as the effect estimates for polypharmacy on incident frailty were similar to that of the primary definition. Finally, using the all polypharmacy definition as the exposure, we found an adjusted OR of 3.03 (95% CI 1.52–6.05) of incident frailty for the individuals with hyperpolypharmacy compared to those with no polypharmacy.

### Modification of the effect of polypharmacy on incident frailty by CKD

Individuals with CKD were older (mean 83.7 vs. 80.5 years) and had a higher CCI (5 [3–8] vs. 4 [2–6]) compared to individuals without CKD (Supplement Table 6). During the follow-up period, independent of their polypharmacy status, only very few individuals without CKD became frail (7.8%) compared to individuals with CKD (19.6%). Individuals with CKD and polypharmacy became more often frail (26.3%) compared to individuals without polypharmacy and CKD (14.1%) (Table 3, Supplement Table 7). In individuals without CKD there was no major difference in frailty incidence between those with (8.7%) and without (7.4%) polypharmacy.

The modification of the effect of polypharmacy on incident frailty by CKD is shown in Table 3 and Supplement Table 8. A significant interaction between CKD and Polypharmacy was observed on an additive scale (RERI 1.56; 95% CI 0.01–3.12) but not on a multiplicative scale (Ratio of ORs 2.34; 95% CI 0.61–9.01) for incident frailty. This demonstrates that the combined effect of polypharmacy and CKD on incident frailty was larger than the sum of the individual effects of polypharmacy and CKD.

**Table 3:** Modification of the effect of polypharmacy on incident frailty by CKD

		No Polypharmacy					Polypharmacy			OR (95% CI) for polypharmacy within the strata of CKD*
		Total	Number of participants with incident frailty (%)	OR (95% CI)			Total	Number of participants with incident frailty (%)	OR (95% CI)	
no CKD		121	9 (7.4)	Reference			46	4 (8.7)	0.95 (0.27, 3.36)	0.95 (0.27, 3.36)
CKD		263	37 (14.1)	1.23 (0.54, 2.79)			198	52 (26.3)	2.74 (1.19, 6.33)	2.23 (1.32, 3.78)
Measure of effect modification on the multiplicative scale:					Ratio of ORs (95% CI): 2.34 (0.61, 9.01)					
Measure of effect modification on the additive scale:					RERI (95% CI): 1.56 (0.01, 3.12)					

CKD: Chronic kidney disease defined as an estimated glomerular filtration rate based on the BIS2 equation < 60 mL/min/1.73m^2^ and/or albuminuria defined as an albumin-creatinine ratio ≥ 30 mg/g; Polypharmacy defined as taking ≥ 5 drugs of regular prescription drugs (regular prescription polypharmacy); RERI: Relative excess risk due to interaction; OR: Odds ratio; 95% CI: 95% Confidence interval

*column represents results from the stratified analyses by CKD with the reference categories being no polypharmacy in each stratum of CKD

All ORs are adjusted for age, gender, short version of the Comparative Analysis of Social Mobility in Industrial Nations classification of education, marital status, Body mass index, Charlson comorbidity index, and smoking

## Discussion

In this cohort of older adults with a mean age of 82.9 years, about 40% were taking at least five prescribed drugs regularly. Among those with regular prescription polypharmacy and hyperpolypharmacy, the adjusted OR of incident frailty within the next two years were 1.96 (95% CI 1.20–3.19) and 2.87 (95% CI 0.85–9.69) compared to individuals without polypharmacy respectively. Using extended polypharmacy definitions, the association with incident frailty was attenuated for polypharmacy. The effect of polypharmacy on incident frailty was modified by CKD on an additive scale (RERI 1.56; 95% CI 0.01, 3.12).

The polypharmacy prevalence of 40% found in our study is comparable to the prevalence in the age group ≥ 75 years in many European countries as assessed by the Survey of Health, Ageing, and Retirement in Europe (SHARE) [37]. The most prevalent medication categories at the therapeutic subgroup level are similar to those reported for a community-based cohort study in the United States and across multiple European countries, likely because they reflect the major chronic diseases in older adults [13, 38]. This probably explains why the top ten medication groups and the five most prescribed substances within the groups did not differ between polypharmacy categories although the prevalence of each substance varied across categories.

A meta-analysis on global incidence of frailty among community-dwelling older adults found that among non-frail individuals who survived a median (IQR) of 3.0 (1.0-11.7) years, 13.6% became frail [39]. In our study, 16.2% (105 of 649 participants who survived the follow-up period) became frail within a median follow-up of 2.1 years. Since it has been shown that frailty incidence increases with age [9], the higher cumulative incidence in our study could be explained by the older age of our study participants.

Longitudinal studies that investigated the association of polypharmacy and incident frailty in older adults were inconclusive. Some found that polypharmacy increased the risk of incident frailty [8, 9, 12] while others demonstrated no association [10, 11, 13]. One possible explanation may be different definitions of polypharmacy. Our results show that older adults with polypharmacy have almost double the odds for incident frailty compared to individuals without polypharmacy. The estimated effect decreases when the definition of polypharmacy is expanded to include on-demand drugs, OTC drugs, vitamins and supplements and the effect becomes more prominent in the hyperpolypharmacy group as the definition expands. Our results using the active substance polypharmacy definition are comparable to another study that used that same definition but with younger individuals (mean age 70 years) and three years of follow-up [9]. Another study with a two-year follow-up of older men (mean age of 77 years) used our primary polypharmacy definition (regular prescription) and found for individuals with hyperpolypharmacy a 2.5 odds of incident frailty [8] which is comparable to our findings. Studies that did not find an association between polypharmacy and incident frailty used polypharmacy definitions including a lower cut-off (three medications and not five) [11], assessed medication on a linear scale [10], or used an all polypharmacy definition and a longer follow-up [12, 13]. Shmuel et al. additionally included a regular and on-demand polypharmacy definition and also found a non-significant OR of 1.4 (95% CI 0.9-2.0) of incident frailty for individuals with polypharmacy comparable to our study (OR: 1.46; 95% CI 0.89–2.40) [13]. Thus, when investigating the risk of polypharmacy on incident frailty in older adults, it is important to note that the use of different definitions of polypharmacy may result in different effect estimates of the impact of polypharmacy on incident frailty.

Since the association of polypharmacy and incident frailty remained significant after adjusting for comorbidities, other possible pathways independent of comorbidities may exist in which polypharmacy could lead to frailty. (1) Overall, polypharmacy increases the risk of taking potentially inappropriate medications, adverse drug events, and low adherence, all of which are also associated with frailty [40]. (2) Polypharmacy and specific drug classes (e.g., acetylcholinesterase inhibitors and HMG-CoA reductase inhibitors) can lead to weight loss, malnutrition, and sarcopenia through alterations in taste, intestinal absorption and metabolism, or elimination of vitamins and minerals, which in turn reflect important components of the fraily phenotype [5, 41]. (3) Another important aspect of the frailty phenotype is physical activity. It has been shown that an increasing number of medications and polypharmacy is associated with decreased physical activity [42]. This pathway operates probably via specific medications such as statins which are known to be associated with myalgias that could lead to less physical activity [42]. (4) It has also been shown that polypharmacy is associated with slowness, a third component of the frailty phenotype [43].

As CKD is common in old age, it is frequently accompanied by the intake of several drugs and has also been associated with incident frailty [16, 44]. Therefore, we also investigated CKD as a modifier of the effect of polypharmacy on incident frailty. Our results provide strong indications that the estimated effect of polypharmacy on incident frailty is modified by CKD on the additive scale. It has been argued that analyzing the biological interaction on an additive rather than a multiplicative scale is the appropriate approach in public health [34]. This implies that the combined effect of polypharmacy and CKD on incident frailty is larger than the sum of the individual effects [45]. A possible explanation for the effect modification could be that several potential pathways in which polypharmacy contributes to frailty are similar to those in which CKD can lead to frailty. For example, as described before, polypharmacy can lead to malnutrition [41]. Malnutrition is common in individuals with CKD and may also further decrease kidney function and lead to frailty worsening [46]. Another possible explanation could be that in older adults the drug metabolism and clearance may change especially in individuals with reduced kidney function and the risk of adverse drug reactions is higher in older individuals with CKD [47].

This emphasizes that the consequences of polypharmacy especially in old age are multifaceted. One part of the problem is certainly that guideline treatment decisions are often based on results from clinical trials where older adults, especially with multimorbidity are excluded [48]. Thus, the grounds on which guideline-adherent treatment decisions were made did not include the population in which they are then applied [49]. Furthermore, guidelines are often focused on a single disease [48]. When multimorbidity is treated in older adults this in turn contributes to polypharmacy. Although polypharmacy is recognized as a risk factor for adverse events, it is very prevalent in older adults [50]. Another aspect that should be considered in older adults are the trade-offs between future risk reductions and the potential current risks for adverse events due to polypharmacy [51]. Both the general practitioner and the older patient have to balance risk and benefit from deprescribing carefully [52, 53], particularly since studies showing improvement in clinical outcomes are scarce [2]. A positive development in terms of clinical outcomes are the new drugs such as sodium-glucose cotransporters and steroidal mineralocorticoid receptor antagonists for reducing albuminuria. These could also have a preventive impact, e.g. lowering the risk of frailty.

Our study has several strengths. We have a very old study population with a longitudinal design that is phenotyped in much detail which enabled us to investigate incident frailty. Due to the complimentary linkage of primary (BIS) and secondary (AOK claims) data, it was possible to have available information on an extensive range of different valid health indicators. In addition, a comprehensive assessment of all medications, including packaging and medication schedules, was conducted. This allowed the inclusion of not only prescription but also OTC drugs. This detailed medication phenotyping of participants added to the strength of the study and enabled different polypharmacy definitions to be compared. Furthermore, we applied the methodological approach proposed by Knol and VanderWeele to analyze and demonstrate effect modification not only on the multiplicative but also on the additive scale to assess its public health importance [33].

The study has some limitations. The assessment of polypharmacy was based on self-reported medication use which is prone to recall bias, supported by medication plans and packages when available. However, we could not determine the number of pills, the dosage, or the extent of medication adherence. We also did not consider whether medications were appropriate for the severity and progression of disease, although this may be important in assessing frailty risk [8]. We did, however, consider the number of morbidities using a comorbidity score (CCI) which includes weights for comorbidity severity but does not take individual disease progression into account. Adjustment for comorbidities also served to address confounding by indication. Although we adjusted for multiple confounders we cannot exclude residual confounding. On the other hand, the research question addressed in this study could not have been answered in a randomized controlled trial, as it is neither ethically acceptable to assign an individual to polypharmacy if it is not indicated nor is it even possible to initiate CKD in individuals and consequently randomly assign them. A further possible limitation could be that we only assessed CKD at baseline; however, our CKD definition was consistent with the KDIGO aspect on chronicity [29] in at least 97% of the participants, thus technically justifying the use of the term CKD. Another aspect is loss to follow-up during the observation period that could have led to selection bias. The loss to follow-up seems to be independent of the exposure since it is evenly distributed across the exposure categories. Frailty incidence is cumulative as we analyzed frailty over two time points. Since frailty is a dynamic process, we cannot exclude that we missed transitions e.g. from frailty to non-frailty / prefrailty.

## Conclusions

In conclusion, our study demonstrates the importance of the polypharmacy definition when estimating incident frailty and provide strong evidence for an effect modification of CKD on polypharmacy and incident frailty on an additive scale which has important public health implications. A target to prevent the occurrence of frailty could be the revision of prescription medications, especially in older patients with CKD.

### Electronic supplementary material

Below is the link to the electronic supplementary material.

Supplementary Material 1

## Data Availability

There are no linked research datasets for this study. The data are available from the corresponding author OR from the study PI upon reasonable request.

## Abbreviations

ACR: Albumin-creatinine ratio
AOK: Statutory health insurance fund *AOK Nordost– Die Gesundheitskasse*
ATC: Anatomical Therapeutic Chemical
BIS: Berlin Initiative Study
CASMIN: Comparative Analysis of Social Mobility in Industrial Nations
CCI: Charlson Comorbidity Index
CI: Confidence interval
CKD: Chronic kidney disease
eGFR: Estimated glomerular filtration rate
ICD-10: 10th Revision of the International Statistical Classification of Diseases and Related Health Problems
IQR: Interquartile range
OR: Odds ratio
OTC: Over-the-counter
RERI: Relative Excess Risk due to Interaction
SD: Standard deviation
SHARE: Survey of Health, Ageing, and Retirement in Europe
STROBE: Strengthening the Reporting of Observational Studies in Epidemiology
