# Supplementary material for: Effect modification of polypharmacy on incident frailty by chronic kidney disease in older adults
Source: BMC Geriatr. 2024 Apr 12;24:335. doi: 10.1186/s12877-024-04887-5 (PMC11015642; doi:10.1186/s12877-024-04887-5)
Supplement: Supplementary file 1 — Supplementary Material 1 [file 12877_2024_4887_MOESM1_ESM.docx]

**Effect modification of Polypharmacy on incident Frailty by Chronic Kidney Disease in older adults**

**Supplement**

**A Determination of kidney disease chronicity**

**B STROBE Statement**

**Supplement Table 1:** Prevalence (%) of the five most frequently prescribed drugs on the 7-digit ATC level for the identified top ten 3-digit ATC drug groups stratified by regular prescription polypharmacy

**Supplement Table 2:** Death before follow-up stratified by polypharmacy status

**Supplement Table 3:** Death before follow-up stratified by polypharmacy and CKD status

**Supplement Table 4:** Main characteristics of the non-frail study population by inclusion status at baseline

**Supplement Table 5:** Sensitivity analyses for the association of dichotomous polypharmacy definitions and incident frailty

**Supplement Table 6:** Main characteristics of the non-frail study population by regular prescription polypharmacy stratified by CKD status at baseline

**Supplement Table 7:** Association of polypharmacy with incident frailty stratified by CKD status

**Supplement Table 8:** Modification of the effect of polypharmacy on incident frailty by CKD (interaction term)

**Supplement A Determination of kidney disease chronicity**

According to KDIGO guideline*, CKD is defined as abnormalities of kidney structure or function, present for more than 3 months. To confirm that our CKD definition complies with the KDIGO one, individuals with an eGFR-BIS2 <60 mL/min/1.73 m^2^ or ACR ≥30 mg/g before and after frailty baseline were analyzed to show how many participants were already in or remained in these categories. This could be shown in 97.4% of participants, thus technically justifying the use of the term CKD.

*Kidney Disease: Improving Global Outcomes (KDIGO) CKD Work Group. KDIGO 2012 Clinical Practice Guideline for the Evaluation and Management of Chronic Kidney Disease. Kidney International Supplements. 2013;3(1):1-150.

**Supplement B**

STROBE Statement—Checklist of items that should be included in reports of ***cohort studies***

|  | **Item No** | | | **Recommendation** | **Page No** |  |
| --- | --- | --- | --- | --- | --- | --- |
| **Title and abstract** | 1 | | | (*a*) Indicate the study’s design with a commonly used term in the title or the abstract | Abstract |  |
|  |  |  |  | (*b*) Provide in the abstract an informative and balanced summary of what was done and what was found | Abstract |  |
| **Introduction** | | | | | |  |
| Background/ rationale | 2 | | | Explain the scientific background and rationale for the investigation being reported | Introduction |  |
| Objectives | 3 | | | State specific objectives, including any prespecified hypotheses | Introduction, last paragraph |  |
| **Methods** | | | | | |  |
| Study design | 4 | | | Present key elements of study design early in the paper | Methods – Study population |  |
| Setting | 5 | | | Describe the setting, locations, and relevant dates, including periods of recruitment, exposure, follow-up, and data collection | Methods – Study population |  |
| Participants | 6 | | | (*a*) Give the eligibility criteria, and the sources and methods of selection of participants. Describe methods of follow-up | Methods – Study population |  |
|  |  |  |  | (*b*) For matched studies, give matching criteria and number of exposed and unexposed | Not applicable |  |
| Variables | 7 | | | Clearly define all outcomes, exposures, predictors, potential confounders, and effect modifiers. Give diagnostic criteria, if applicable | Methods - Exposure: polypharmacy assessment, Outcome: incident frailty and Covariable assessment |  |
| Data sources/ measurement | 8* | | | For each variable of interest, give sources of data and details of methods of assessment (measurement). Describe comparability of assessment methods if there is more than one group | Methods - Covariable assessment |  |
| Bias | 9 | | | Describe any efforts to address potential sources of bias |  |  |
| Study size | 10 | | | Explain how the study size was arrived at | Methods – Study population |  |
| Quantitative variables | 11 | | | Explain how quantitative variables were handled in the analyses. If applicable, describe which groupings were chosen and why | Methods –Statistical  analyses |  |
| Statistical methods | 12 | | | (*a*) Describe all statistical methods, including those used to control for confounding | Methods –Statistical  analyses |  |
|  |  |  |  | (*b*) Describe any methods used to examine subgroups and interactions |  |  |
|  |  |  |  | (*c*) Explain how missing data were addressed |  |  |
|  |  |  |  | (*d*) If applicable, explain how loss to follow-up was addressed |  |  |
|  |  |  |  | (*e*) Describe any sensitivity analyses |  |  |
| **Results** | | | | |  |  |
| Participants | 13* | | | (a) Report numbers of individuals at each stage of study—eg numbers potentially eligible, examined for eligibility, confirmed eligible, included in the study, completing follow-up, and analysed | Figure 1 |  |
|  |  |  |  | (b) Give reasons for non-participation at each stage |  |  |
|  |  |  |  | (c) Consider use of a flow diagram |  |  |
| Descriptive data | 14* | | | (a) Give characteristics of study participants (eg demographic, clinical, social) and information on exposures and potential confounders | Results – Table 1 |  |
|  |  |  |  | (b) Indicate number of participants with missing data for each variable of interest |  |  |
|  |  |  |  | (c) Summarise follow-up time (eg, average and total amount) |  |  |
| Outcome data | 15* | | | Report numbers of outcome events or summary measures over time | Results – Polypharmacy and incident frailty |  |
| Main results | | 16 | (*a*) Give unadjusted estimates and, if applicable, confounder-adjusted estimates and their precision (eg, 95% confidence interval). Make clear which confounders were adjusted for and why they were included | | Results – Polypharmacy and incident frailty, Table 2 | |
|  |  |  | (*b*) Report category boundaries when continuous variables were categorized | |  | |
|  |  |  | (*c*) If relevant, consider translating estimates of relative risk into absolute risk for a meaningful time period | |  | |
| Other analyses | | 17 | Report other analyses done—eg analyses of subgroups and interactions, and sensitivity analyses | | Results – Effect estimation using different polypharmacy definitions and Modification of the effect of polypharmacy on incident frailty by CKD | |
| **Discussion** | | | | | |  |
| Key results | | 18 | Summarise key results with reference to study objectives | | Discussion – 1st  paragraph | |
| Limitations | | 19 | Discuss limitations of the study, taking into account sources of potential bias or imprecision. Discuss both direction and magnitude of any potential bias | | Discussion – before  last paragraph | |
| Interpretation | | 20 | Give a cautious overall interpretation of results considering objectives, limitations, multiplicity of analyses, results from similar studies, and other relevant evidence | | Discussion | |
| Generalisability | | 21 | Discuss the generalisability (external validity) of the study results | | Discussion | |
| **Other information** | | | | | |  |
| Funding | | 22 | Give the source of funding and the role of the funders for the present study and, if applicable, for the original study on which the present article is based | | Funding paragraph | |

*Give information separately for exposed and unexposed groups.

**Note:** An Explanation and Elaboration article discusses each checklist item and gives methodological background and published examples of transparent reporting. The STROBE checklist is best used in conjunction with this article (freely available on the Web sites of PLoS Medicine at http://www.plosmedicine.org/, Annals of Internal Medicine at http://www.annals.org/, and Epidemiology at http://www.epidem.com/). Information on the STROBE Initiative is available at http://www.strobe-statement.org.

**Supplement Table 1:** **Prevalence (%) of the five most frequently prescribed drugs on the 7-digit ATC level for the identified top ten 3-digit ATC drug groups stratified by regular prescription polypharmacy**

| **3-digit ATC** | **No Polypharmacy (N=)** | | **Polypharmacy (N=)** | | **Hyperpolypharmacy (N=)** | |
| --- | --- | --- | --- | --- | --- | --- |
|  | **Drug** | **Prevalence (%)** | **Drug** | **Prevalence (%)** | **Drug** | **Prevalence (%)** |
| **C09** | Ramipril | 16.6 | Ramipril | 26.1 | Ramipril | 36.4 |
|  | Candesartan | 8.1 | Candesartan | 12.0 | Candesartan | 22.7 |
|  | Valsartan | 7.0 | Valsartan | 11.6 | Valsartan | 13.6 |
|  | Enalapril | 3.3 | Enalapril | 9.8 | Irbesartan | 9.1 |
|  | Telmisartan | 2.6 | Lisinopril | 4.4 | Candesartan and Hydrochlorothiazide | 4.6 |
| **C07** | Metoprolol | 16.6 | Metoprolol | 33.7 | Metoprolol | 54.6 |
|  | Bisoprolol | 12.0 | Bisoprolol | 26.1 | Bisoprolol | 27.3 |
|  | Nebivolol | 1.3 | Nebivolol | 4.0 | Nebivolol | 9.1 |
|  | Carvedilol | 1.1 | Atenolol | 1.8 | Carvedilol | 4.6 |
|  | Propranolol | 0.7 | Carvedilol | 1.8 | Propranolol | 4.6 |
| **C10** | Simvastatin | 17.2 | Simvastatin | 44.6 | Simvastatin | 50.0 |
|  | Atorvastatin | 3.1 | Atorvastatin | 9.8 | Atorvastatin | 18.2 |
|  | Ezetimib | 0.7 | Fluvastatin | 3.3 | Pravastatin | 9.1 |
|  | Fluvastatin | 0.4 | Pravastatin | 2.9 | Fluvastatin | 4.6 |
|  | Pravastatin | 0.4 | Ezetimib | 1.1 | Omega-3-triglycerides incl. other esters and acids | 4.6 |
| **H03** | Levothyroxine Sodium | 14.2 | Levothyroxine Sodium | 24.6 | Levothyroxine Sodium | 54.6 |
|  | Levothyroxine sodium and iodine compounds | 0.7 | Levothyroxine sodium and iodine compounds | 1.8 | n.a. | - |
|  | Combination of Levothyroxine and Liothyronine | 0.2 | Thiamazole | 1.5 | n.a. | - |
|  | Thiamazole | 0.2 | n.a. | - | n.a. | - |
|  | n.a. | - | n.a. | - | n.a. | - |
| **C08** | Amlodipine | 8.3 | Amlodipine | 22.1 | Amlodipine | 40.9 |
|  | Lercanidipine | 3.1 | Lercanidipine | 8.3 | Lercanidipine | 9.1 |
|  | Nitrendipine | 0.7 | Felodipine | 2.9 | Nifedipine | 9.1 |
|  | Verapamil | 0.7 | Verapamil | 2.5 | Felodipine | 4.6 |
|  | Felodipine | 0.4 | Nifedipine | 1.8 | Nitrendipine | 4.6 |
| **B01** | Phenprocoumon | 5.0 | Phenprocoumon | 17.0 | Rivaroxaban | 27.3 |
|  | Apixaban | 3.1 | Rivaroxaban | 6.9 | Phenprocoumon | 22.7 |
|  | Rivaroxaban | 2.8 | Clopidogrel | 4.7 | Clopidogrel | 18.2 |
|  | Clopidogrel | 1.1 | Apixaban | 4.0 | n.a. | - |
|  | Dabigatranetexilat | 0.9 | Edoxaban | 1.5 | n.a. | - |
| **C03** | Torasemide | 4.4 | Torasemide | 25.0 | Torasemide | 63.6 |
|  | Hydrochlorothiazide | 3.9 | Hydrochlorothiazide | 17.0 | Eplerenon | 9.1 |
|  | Spironolactone | 1.1 | Spironolactone | 7.3 | Hydrochlorothiazide | 9.1 |
|  | Hydrochlorothiazide and Triamteren | 0.7 | Furosemide | 3.3 | Furosemide | 4.6 |
|  | Spironolacton and  Furosemide | 0.7 | Xipamide | 1.1 | Hydrochlorothiazide and Triamteren | 4.6 |
| **G04** | Tamsulosin | 5.7 | Tamsulosin | 10.5 | Tamsulosin | 9.1 |
|  | Trospium | 1.1 | Tamsulosin and Dutasteride | 3.6 | Propiverin | 4.6 |
|  | Finasteride | 0.9 | Finasteride | 2.9 | Solifenacin | 4.6 |
|  | Silodosin | 0.7 | Alfuzosin | 0.7 | Trospium | 4.6 |
|  | Dutasteride | 0.4 | Duloxetin | 0.7 | n.a. | - |
| **A02** | Pantoprazole | 6.8 | Pantoprazole | 22.1 | Pantoprazole | 36.4 |
|  | Omeprazole | 1.7 | Omeprazole | 5.8 | Omeprazole | 13.6 |
|  | Esomeprazole | 1.3 | Esomeprazole | 4.0 | Esomeprazole | 4.6 |
|  | Ranitidine | 0.2 | Ranitidine | 1.1 | Ranitidine | 4.6 |
|  | Sucralfate | 0.2 | Famotidine | 0.4 | n.a. | - |
| **A10** | Metformin | 4.1 | Metformin | 18.1 | Insulin (human) | 27.3 |
|  | Glimepiride | 1.5 | Insulin (human) | 9.4 | Metformin | 18.2 |
|  | Sitagliptin | 1.1 | Sitagliptin | 6.5 | Insulin glargin | 13.6 |
|  | Insulin (human) | 0.4 | Glimepiride | 5.4 | Sitagliptin | 13.6 |
|  | Glibenclamide | 0.2 | Insulin glargin | 3.6 | Glibenclamide | 4.6 |

n.a. - not applicable: no further drugs on the chemical substance level

Multiple drugs for each participant are possible.

**Supplement Table 2: Death before follow-up stratified by polypharmacy status**

|  | **Total** | **No Polypharmacy** | **Polypharmacy** | **Hyperpolypharmacy** |
| --- | --- | --- | --- | --- |
| n | 757 | 459 | 276 | 22 |
| Died before the follow-up visit, n (%) | 37 (4.9) | 18 (3.9) | 16 (5.8) | 3 (13.6) |

**Supplement Table 3: Death before follow-up stratified by polypharmacy and CKD status**

|  | **CKD** | | | | **No CKD** | | | | |
| --- | --- | --- | --- | --- | --- | --- | --- | --- | --- |
|  | **Total** | **No Polypharmacy** | **Polypharmacy** | **Hyperpolypharmacy** | **Total** | **No Polypharmacy** | **Polypharmacy** | **Hyperpolypharmacy** |  |
| n | 557 | 312 | 224 | 21 | 189 | 139 | 49 | 1 |  |
| Died before the follow-up visit, n (%) | 33 (5.9) | 14 (4.5) | 16 (7.1) | 3 (14.3) | 4 (2.1) | 4 (2.9) | 0 | 0 |  |

**Supplement Table 4: Main characteristics of the non-frail study population by inclusion status at baseline**

|  | **Total** | **Excluded** | **Included** |
| --- | --- | --- | --- |
| n | 757 | 121 | 636 |
| Age in years, mean (SD) | 82.9 (4.9) | 84.4 (5.5) | 82.6 (4.7) |
| Gender, n (%)  Female | 397 (52.4) | 59 (48.8) | 338 (53.1) |
| Polypharmacy status, n (%) |  |  |  |
| No polypharmacy | 459 (60.6%) | 70 (57.9%) | 389 (61.2%) |
| Polypharmacy | 276 (36.5%) | 47 (38.8%) | 229 (36.0%) |
| Hyperpolypharmacy | 22 (2.9%) | 4 (3.3%) | 18 (2.8%) |
| Frailty status, n (%) |  |  |  |
| Robust | 225 (29.7%) | 31 (25.6%) | 194 (30.5%) |
| Pre-frail | 532 (70.3%) | 90 (74.4%) | 442 (69.5%) |
| CASMIN, n (%) |  |  |  |
| Low | 450 (59.4) | 70 (57.9) | 380 (59.7) |
| Middle | 147 (19.4) | 22 (18.2) | 125 (19.7) |
| High | 157 (20.7) | 26 (21.5) | 131 (20.6) |
| Missing | 3 (0.4) | 3 (2.5) | 0 |
| Marital Status, n (%) |  |  |  |
| Married | 395 (52.2) | 55 (45.5) | 340 (53.5) |
| Single | 35 (4.6) | 8 (6.6) | 27 (4.2) |
| Divorced | 62 (8.2) | 11 (9.1) | 51 (8.0) |
| Widowed | 265 (35.0) | 47 (38.8) | 218 (34.3) |
| Smoking, n (%) |  |  |  |
| Ever | 354 (46.8) | 62 (51.2) | 292 (45.9) |
| BMI in kg/m^2^, n (%) |  |  |  |
| <22 | 72 (9.5) | 11 (9.1) | 61 (9.6) |
| 22-<30 | 535 (70.7) | 88 (72.7) | 447 (70.3) |
| ≥30 | 150 (19.8) | 22 (18.2) | 128 (20.1) |
| CCI, median [IQR] | 5 [3-7] | 6 [3-9] | 5 [3-7] |
| Missing, n (%) | 11 (1.5) | 5 (4.1) | 0 |
| CKD, n (%) | 557 (73.6) | 96 (79.3) | 461 (72.5) |
| Missing | 11 (1.5) | 3 (2.5) | 8 (1.3) |
| eGFR_BIS2_ |  |  |  |
| mean (SD) | 52.9 (12.6) | 49.7 (14.4) | 53.5 (12.1) |
| Missing, n (%) | 4 (0.5) | 2 (1.7) | 2 (0.3) |
| ACR, n (%) |  |  |  |
| ≥30 mg/g | 176 (23.2) | 41 (33.9) | 135 (21.2) |
| Missing | 19 (2.5) | 4 (3.3) | 15 (2.4) |

BMI: Body mass index; CCI: Charlson Comorbidity Index, CKD: Chronic kidney disease defined as eGFR_BIS2_ <60 mL/min/1.73m^2^ and/or albuminuria defined as ACR ≥30 mg/g.; eGFR_BIS2_: Estimated glomerular filtration rate based on the BIS2 equation; ACR: Albumin-creatinine ratio; CASMIN: short version of the Comparative Analysis of Social Mobility in Industrial Nations classification of education; IQR: Interquartile range; No missing values for age, gender, polypharmacy status, Frailty status, marital status, smoking, BMI

**Supplement Table 5: Sensitivity analyses for the association of dichotomous polypharmacy definitions and incident frailty**

|  | **Total** | **Number of participants with incident frailty (%)** | **Crude Model**  **OR (95% CI)** | **Adjusted Model***  **OR (95% CI)** |
| --- | --- | --- | --- | --- |
| **Regular Prescription Polypharmacy** |  |  |  |  |
| No Polypharmacy | 389 | 46 (11.8) | Reference | Reference |
| Polypharmacy | 247 | 56 (22.7) | 2.19 (1.43 – 3.36) | 1.99 (1.23 – 3.23) |
|  |  |  |  |  |
| **Active Substance Polypharmacy** |  |  |  |  |
| No Polypharmacy | 367 | 44 (12.0) | Reference | Reference |
| Polypharmacy | 269 | 58 (21.6) | 2.02 (1.32 – 3.10) | 1.81 (1.12 – 2.91) |
|  |  |  |  |  |
| **Regular Polypharmacy** |  |  |  |  |
| No Polypharmacy | 319 | 39 (12.2) | Reference | Reference |
| Polypharmacy | 317 | 63 (19.9) | 1.78 (1.15 – 2.75) | 1.46 (0.89 – 2.40) |
|  |  |  |  |  |
| **Regular and On-Demand Polypharmacy** |  |  |  |  |
| No Polypharmacy | 261 | 29 (11.1) | Reference | Reference |
| Polypharmacy | 375 | 73 (19.5) | 1.93 (1.22 – 3.07) | 1.72 (1.02 – 2.90) |
|  |  |  |  |  |
| **All Polypharmacy** |  |  |  |  |
| No Polypharmacy | 207 | 22 (10.6) | Reference | Reference |
| Polypharmacy | 429 | 80 (18.6) | 1.93 (1.16 – 3.19) | 1.73 (0.99 – 3.02) |

OR: odds ratio; 95% CI: 95% confidence interval; *regular prescription polypharmacy:* number of regular prescription drugs; *active substance polypharmacy:*number of active substances in regular prescription drugs accounting for more than one active substance in combination drugs; *regular polypharmacy*: number of regular prescription and regular OTC drugs; *regular and on-demand polypharmacy*: additionally including the number of on-demand prescription and on-demand OTC drugs; *all polypharmacy:* all drugs including the number of vitamins, minerals, and supplements. For all definitions, no polypharmacy was defined as taking of 0-4 drugs, and polypharmacy as taking of ≥5 drugs.

*Adjusted for age, gender, smoking, short version of the Comparative Analysis of Social Mobility in Industrial Nations classification of education, marital status, Charlson Comorbidity Index, and Body mass index

**Supplement Table 6: Main characteristics of the non-frail study population by regular prescription polypharmacy stratified by CKD status at baseline**

|  | **CKD** | | | | **No CKD** | | | | |
| --- | --- | --- | --- | --- | --- | --- | --- | --- | --- |
|  | **Total** | **No Polypharmacy** | **Polypharmacy** | **Hyperpolypharmacy** | **Total** | **No Polypharmacy** | **Polypharmacy** | **Hyperpolypharmacy** |  |
| n | 557 | 312 | 224 | 21 | 189 | 139 | 49 | 1 |  |
| Age in years, mean (SD) | 83.7 (5.1) | 84.1 (5.3) | 83.3 (4.9) | 81.8 (3.7) | 80.5 (3.2) | 80.4 (3.3) | 80.8 (3.0) | 79.3 |  |
| Gender, n (%) |  |  |  |  |  |  |  |  |  |
| Female | 288 (51.7) | 165 (52.9) | 112 (50.0) | 11 (52.4) | 100 (52.9) | 78 (56.1) | 21 (42.9) | 1 (100) |  |
| CASMIN, n (%) |  |  |  |  |  |  |  |  |  |
| Low | 331 (59.4) | 180 (57.7) | 138 (61.6) | 13 (61.9) | 110 (58.2) | 76 (54.7) | 33 (67.3) | 1 (100) |  |
| Middle | 95 (17.1) | 54 (17.3) | 38 (17.0) | 3 (14.3) | 51 (27.0) | 40 (28.8) | 11 (22.4) | 0 |  |
| High | 128 (23.0) | 77 (24.7) | 46 (20.5) | 5 (23.8) | 28 (14.8) | 23 (16.5) | 5 (10.2) | 0 |  |
| Missing | 3 (0.5) | 1 (0.3) | 2 (0.9) | 0 | 0 | 0 | 0 | 0 |  |
| Marital Status, n (%) |  |  |  |  |  |  |  |  |  |
| Married | 275 (49.4) | 153 (49.0) | 108 (48.2) | 14 (66.7) | 113 (56.8) | 77 (55.4) | 35 (71.4) | 1 (100) |  |
| Single | 32 (5.7) | 21 (6.7) | 11 (4.9) | 0 | 2 (1.1) | 1 (0.7) | 1 (2.0) | 0 |  |
| Divorced | 40 (7.2) | 25 (8.0) | 15 (6.7) | 0 | 21 (11.1) | 19 (13.7) | 2 (4.1) | 0 |  |
| Widowed | 210 (37.7) | 113 (36.2) | 90 (40.2) | 7 (33.3) | 53 (28.0) | 42 (30.2) | 11 (22.4) | 0 |  |
| Smoking, n (%) |  |  |  |  |  |  |  |  |  |
| Ever | 270 (48.5) | 140 (44.9) | 115 (51.3) | 15 (71.4) | 82 (43.4) | 58 (41.7 | 24 (49.0) | 0 |  |
| BMI in kg/m^2^, n (%) |  |  |  |  |  |  |  |  |  |
| <22 | 46 (8.3) | 36 (11.5) | 10 (4.5) | 0 | 24 (12.7) | 19 (13.7) | 5 (10.2) | 0 |  |
| 22-<30 | 386 (69.3) | 218 (69.9) | 152 (67.9) | 16 (76.2) | 142 (75.1) | 106 (76.3) | 35 (71.4) | 1 (100) |  |
| ≥30 | 125 (22.4) | 58 (18.6) | 62 (27.7) | 5 (23.8) | 23 (12.2) | 14 (10.1) | 9 (18.4) | 0 |  |
| CCI, median [IQR] | 5 [3-8] | 4 [3-6] | 7 [5-9] | 10 [8-11] | 4 [2-6] | 3 [2-5] | 6 [4-7] | 4 |  |
| Missing, n (%) | 4 (0.7) | 2 (0.6) | 2 (0.9) | 0 | 7 (3.7) | 6 (4.3) | 1 (2) | 0 |  |
| eGFR_BIS2_, mean (SD) | 48.0 (10.6) | 50.2 (9.8) | 45.6 (10.8) | 38.9 (11.2) | 66.6 (5.4) | 66.7 (5.3) | 66.3 (5.7) | 71.9 |  |
| Missing, n (%) | 1 (0.2) | 0 | 0 | 1 (4.8) | 0 | 0 | 0 | 0 |  |
| ACR, median  [IQR] | 12.9  [5.1, 42.0] | 11.7  [4.7, 39.9] | 16.6  [6.2, 44.2] | 13.6  [7.2, 36.6] | 6.5  [4.0, 11.9] | 6.3  [4.0, 11.9] | 7.1  [4.1, 13.0] | 3.2 |  |
| Missing | 8 (1.4%) | 4 (1.3%) | 3 (1.3%) | 1 (4.8%) | 0 | 0 | 0 | 0 |  |

BMI: Body mass index; CCI: Charlson Comorbidity Index, CKD: Chronic kidney disease defined as eGFR_BIS2_ <60 mL/min/1.73m^2^ and/or albuminuria defined as ACR ≥30 mg/g.; eGFR_BIS2_: Estimated glomerular filtration rate based on the BIS2 equation; ACR: Albumin-creatinine ratio; CASMIN: short version of the Comparative Analysis of Social Mobility in Industrial Nations classification of education; IQR: Interquartile range;

No missing values for age, gender, marital status, smoking, BMI

**Supplement Table 7: Association of polypharmacy with incident frailty stratified by CKD status**

|  | **Total** | **Number of participants with incident frailty (%)** | **Crude Model**  **OR (95% CI)** | **Adjusted Model***  **OR (95% CI)** |
| --- | --- | --- | --- | --- |
| **CKD** |  |  |  |  |
|  |  |  |  |  |
| **Regular Prescription Polypharmacy** |  |  |  |  |
| No Polypharmacy | 263 | 37 (14.1) | Reference | Reference |
| Polypharmacy | 198 | 52 (26.3) | 2.18 (1.36 – 3.48) | 2.34 (1.37 – 4.00) |
|  |  |  |  |  |
| **No CKD** |  |  |  |  |
|  |  |  |  |  |
| **Regular Prescription Polypharmacy** |  |  |  |  |
| No Polypharmacy | 121 | 9 (7.4) | Reference | Reference |
| Polypharmacy | 46 | 4 (8.7) | 1.19 (0.35 – 4.06) | 0.71 (0.17 – 3.02) |

OR: odds ratio; 95% CI: 95% confidence interval; *regular prescription polypharmacy:* number of regular prescription drugs (no polypharmacy was defined as taking of 0-4 drugs, and polypharmacy as taking of ≥5 drugs). *Adjusted for age, gender, smoking, short version of the Comparative Analysis of Social Mobility in Industrial Nations classification of education, marital status, Charlson Comorbidity Index, and Body mass index. Total of participants is 628 because 8 are missing CKD status.

**Supplement Table 8: Modification of the effect of polypharmacy on incident frailty by CKD (interaction term)**

|  | **Total** | **Number of participants with incident frailty (%)** | **Crude Model**  **OR (95% CI)** | **Adjusted Model***  **OR (95% CI)** |
| --- | --- | --- | --- | --- |
| **Regular Prescription Polypharmacy** |  |  |  |  |
| No Polypharmacy | 389 | 46 (11.8) | Reference | Reference |
| Polypharmacy | 247 | 56 (22.7) | 1.19 (0.35 – 4.06) | 0.95 (0.27 – 3.37) |
| Polypharmacy x CKD |  |  | 1.84 (0.49 – 6.85) | 2.34 (0.61 – 9.01) |

OR: odds ratio; 95% CI: 95% confidence interval; *regular prescription polypharmacy:* number of regular prescription drugs (no polypharmacy was defined as taking of 0-4 drugs, and polypharmacy as taking of ≥5 drugs). *Adjusted for age, gender, smoking, short version of the Comparative Analysis of Social Mobility in Industrial Nations classification of education, marital status, Charlson Comorbidity Index, and Body mass index. Total of participants is 628 because 8 are missing CKD status.
